# Job exposures, employer characteristics, and risk of reduced work capacity: a 10-year cohort study of Norwegian workers

**DOI:** 10.1007/s00420-025-02195-y

**Published:** 2026-01-03

**Authors:** Julie Ulstein, Cedric Andersen Lyngroth, Åsmund Hermansen

**Affiliations:** 1 Research Group for Occupational Medicine and Epidemiology, National Institute for Occupational Health, Oslo, Norway; 2 Department of Social Work, Child Welfare and Social Policy, Oslo Metropolitan University, Oslo, Norway; 3 Research Group for Work Psychology and Physiology, National Institute of Occupational Health, Oslo, Norway

**Keywords:** Employer characteristics, Job exposures, Job exposure matrices, Biomechanical exposures, Psychosocial exposures, Work capacity

## Abstract

**Objective:**

This study investigates the impact of biomechanical and psychosocial job exposures on risk of reduced work capacity in a complete cohort of Norwegian workers, and examines whether this impact varies by employer sector, size, and organizational policies.

**Methods:**

Using high-quality Norwegian registry data, we followed a cohort of workers from age 40 over a ten-year period. Biomechanical and psychosocial job exposures were estimated using two validated job exposure matrices. Individuals with a prior history of reduced work capacity were excluded to limit confounding. Using Cox proportional hazard models, we assessed the association between levels of job exposure and risk of reduced work capacity, including moderation analyses by employer characteristics.

**Results:**

Both biomechanical and psychosocial job exposures were significantly associated with reduced work capacity, particularly among the top 60% of exposed workers. While employer size and organizational policies somewhat moderated this impact, their influence was inconsistent. Notably, policies aimed at retaining workers with reduced capacity did not appear to mitigate the impact of the job exposures, while there was no variation in impact according to employer sector.

**Conclusion:**

Biomechanical and psychosocial job exposures are associated with an increased risk of reduced work capacity, with some variation in impact according to employer characteristics. These results indicate the importance of exposure-reducing interventions in the workplace, especially in occupations with high levels of biomechanical and psychosocial exposures.

**Supplementary Information:**

The online version contains supplementary material available at 10.1007/s00420-025-02195-y.

## Introduction

Worker health is a critical determinant of a productive and sustainable workforce. At the societal level, a productive workforce is important to sustain the welfare state through tax income (McCollum 2012). For organizations, a sustainable workforce can contribute to a stable supply of skilled labor and limit labor shortages. For individuals, working has generally been viewed as an avenue that can contribute to social integration, economic independence, improved health and wellbeing, and a meaningful life (De witte et al. 2016; Fisher 2010). However, various workplace conditions—particularly biomechanical and psychosocial job exposures—can contribute to the decline of worker health and wellbeing over time (Burgard and Lin 2013; Grzywacz and Dooley 2003; Waddell and Burton 2006). Biomechanical exposures, such as heavy physical labor, and psychosocial exposures, such as limited job control, have been linked to adverse health outcomes that can impair the ability to meet job demands. Over time, such exposures can lead to musculoskeletal disorders or mental health disorders, reducing individual work capacity either temporarily or permanently, depending on the severity of exposure (Ervasti et al. 2019; Niedhammer et al. 2021). Understanding the extent to which biomechanical and psychosocial job exposures can influence work capacity is essential for identifying at-risk workers and developing targeted interventions to promote sustainable employment and prevent premature labor market exits.

Employers play a pivotal role both in recognizing at-risk conditions and implementing workplace strategies to mitigate the impact of job exposures. Factors such as company size, sector, and organizational policies may influence how different job exposures impact individual work capacity. For example, organizations that have implemented preventative policies and practices for worker health and wellbeing may be better equipped to reduce the negative effects of physically and psychologically demanding jobs, while those lacking such policies may see a higher proportion of workers experiencing adverse health outcomes, like reduced work capacity, and temporary or permanent labor market exits. Previous research has demonstrated that these characteristics can impact the employment duration of workers with reduced capacity (Ulstein 2023; Van Ooijen et al. 2021). However, there is still limited research exploring whether—and how—the relationship between job exposures and work capacity varies across different types of employers. This study seeks to address this gap by examining the following research question: *Does the impact of biomechanical and psychosocial job exposures on risk of reduced work capacity vary with employer characteristics?*

Addressing this question can offer valuable insights for employers, policymakers, and occupational health professionals seeking to reduce and prevent negative impacts of job exposures on individual work capacity, contributing to sustainable employment outcomes. By linking validated job exposure matrices (JEMs) with comprehensive Norwegian register data, this study follows a cohort of workers with no prior history of reduced work capacity from 2010 to 2019. Using survival analysis, we estimate the impact of biomechanical and psychosocial job exposures on risk of reduced work capacity and examine whether this impact varies based on employer size, sector, and organizational policies.

### Biomechanical and psychosocial job exposures

Biomechanical job exposures consist of factors such as heavy lifting, awkward working positions, and prolonged standing or walking through the workday. These are factors that over time can result in musculoskeletal discomfort and pain disorders (Hermansen and Dahl 2022). Typical occupations with high biomechanical exposures include jobs in construction, health care, and service jobs. Previous research has established a link between biomechanical job exposures and various adverse health outcomes among the working population. Years of exposure to heavy physical effort at work was strongly associated with disability pension due to musculoskeletal disorders (Ervasti et al. 2019; Kjellberg et al. 2016). Similarly, biomechanical job exposures have been linked to early exit from the labor market, and long-term sickness absence (d’Errico et al. 2021; Sterud 2013). These studies indicate that biomechanical exposures at work can lead to health conditions that reduce individual work capacity.

Psychosocial job exposures, on the other hand, are best understood through Karaseks’ (1979) model on job strain, which include aspects such as psychological demands, decision latitude, and social support. The job strain model posits that a combination of high job-related demands, low control over job-related decision making and low job support can lead to mental strain. Experiencing job strain has been associated with a variety of adverse health outcomes, like mental illness, musculoskeletal symptoms, insomnia, and heart disease (Burgard and Lin 2013; Falkstedt et al. 2023; Niedhammer et al. 2021). These outcomes can reduce individual work capacity and contribute to both temporary and permanent labor market exits through sickness absence or disability benefits.

Building on prior research that demonstrates the adverse impact of both biomechanical and psychosocial job exposures on individual health and labor market participation, the present study investigates whether these associations are moderated by organizational characteristics such as sector, workplace size, and workplace policies and practices. Understanding these moderating factors is critical for identifying points of intervention to improve working conditions and promote sustainable employment.

### Organizational sector

Although research on how sector moderates the relationship between job exposures and worker health is limited, the distinction between public and private employers may still shape both exposure levels and organizational responses. Public sector organizations (e.g. state-owned enterprises, municipalities), typically adhere to stricter regulations and formalized procedures compared to private sector companies (Boyne 2002; Härenstam et al. 2004; Liu and Cheng 2018). These structures can promote consistency and clear protocols for managing working conditions. However, bureaucratic processes and formal communication channels may slow down responses to emerging issues and hinder adjustments to the working environment. Public sector organizations can also be subject to more stringent budget constraints and cost reductions, which can limit organizational opportunities to improve working conditions or invest in accommodation for workers (Härenstam et al. 2004).

In contrast, private sector organizations typically operate with fewer regulatory constraints, leading to greater flexibility in structuring work processes and job tasks. This flexibility can facilitate a more responsive approach to addressing employee needs and workplace challenges (Boyne 2002). Additionally, private employers may face fewer budgetary limitations than their public sector counterparts, potentially allowing for greater investment in improving working conditions (Härenstam et al. 2004). However, less regulation can also result in inconsistencies in workplace policies, leading to local variation in work environments and labor practices. The emphasis on profit generation in private enterprises may limit investments in interventions aimed at improving working conditions, particularly if such measures are not perceived as directly contributing to organizational performance.

Research indicates a negative association between public sector employment and continued labor market participation. For instance, a recent study found stronger links between psychosocial job exposures and sickness absence among young public sector workers—both men and women—compared to their private sector counterparts (Sørensen et al. 2023). Similarly, a Finnish study reported higher rates of full and partial sickness absence in the public sector (Hartikainen et al. 2022). A third study also found that public employment was associated with a higher incidence of disability pensions among women (Albertsen et al. 2007). These findings suggest that biomechanical and psychosocial job exposures may be amplified in the public sector.

### Employer size

Employer size refers to the number of employees in an organization. In the European Union, organizations with fewer than 50 employees are categorized as “small”, while “large” organizations constitute those that employ more than 250 employees (European Commission 2003). The features of large organizations that could impact biomechanical and psychosocial job exposures include substantial resources, presence of human resource professionals, and formalized organizational regulations and practices (Storey et al. 2010). These resources can be used to implement interventions to reduce the impact of job exposures, for example by providing workers with accommodation and follow-up at the workplace, while human resource professionals can play a key role in implementing policies and practices that promote occupational safety (Bacon and Hoque 2021). The broad range of tasks in large organizations can also offer flexibility to workers, allowing for task adjustments, which can contribute to sustainable employment. However, the standardized procedures and uniform practices can reduce opportunities to respond quickly to worker needs and initiate change, and hinder accommodations and interventions necessary to reduce the negative impact of job exposures.

In small organizations, the impact of biomechanical and psychosocial job exposures can be influenced by organizational informality. Characteristics such as informal structures, close interpersonal work environment, and direct communication channels distinguish these workplaces from larger, more bureaucratic institutions (Storey et al. 2010; Tsai et al. 2007). With fewer bureaucratic constraints, small organizations can more easily adjust tasks, workloads, or schedules to address adverse working conditions. This flexibility can enable quicker, tailored interventions that may reduce biomechanical and psychosocial strain. Direct communication can also support the identification and resolution of emerging issues. However, small organizations may also face barriers to systematically addressing job exposures. Limited financial and human resources can make it challenging to implement systematic measures or invest in comprehensive occupational health programs (Champoux and Brun 2003).

Currently, there is no clear evidence on whether the impact of biomechanical and psychosocial job exposures differ by employer size. However, research suggests a negative association for biomechanical exposures, with employees in larger organizations facing lower risks of injury compared to those in smaller workplaces (Fenn and Ashby 2004; Sørensen et al. 2007). In contrast, high job demands, low autonomy, and limited social support—central psychosocial factors—appear more prevalent in large organizations compared to small and medium sized organizations (Knani et al. 2021; Lai et al. 2015; Sørensen et al. 2007). This may suggest that employment in larger organizations helps mitigate the risk associated with biomechanical job exposures, but not psychosocial job exposures.

### Workplace policies and practices

Workplace policies and practices refer to the structured routines, guidelines, and interventions implemented to retain workers with reduced capacity and other vulnerable groups in the workplace (Kersten et al. 2023). The term “implemented” signifies that the policies are actively executed within the organization, ensuring they are followed in practice rather than existing as formal statements. The degree of policy formalization typically varies with firm size and complexity. Smaller organizations often rely on informal or ad hoc approaches, whereas larger organizations tend to adopt formalized rules, regulations, and structured guidelines (Bacon and Hoque 2021). The policies and practices can include measures for support, accommodation, or the adjustment of work hours for workers with reduced capacity. Research suggests a positive association between implemented workplace policies and employment outcomes for vulnerable workers, as organizations with such policies can be more aware of and committed to their responsibilities in workforce retention (Bacon and Hoque 2021).

Empirical findings support these predictions. Workplace policies and practices are linked to higher rates of return to work and continued employment for vulnerable groups, such as workers with reduced capacity (Hulsegge et al. 2022; Kersten et al. 2023; Ulstein 2023; Van Ooijen et al. 2021). Building on previous research, workplace policies and practices may mitigate the impact of biomechanical and psychosocial job exposures by addressing barriers to continued employment and retention for workers with reduced capacity. By strengthening workplace accommodation, support systems, and the overall focus on working conditions, these policies could contribute to sustainable employment and improved work environments for all workers.

### Summary and empirical expectations

Building on the theoretical and empirical background outlined above, we propose four empirical expectations regarding the impact of biomechanical and psychosocial job exposures on individual work capacity and how this impact varies with employer characteristics.

1. Both biomechanical and psychosocial job exposures are expected to be positively associated with the risk of reduced work capacity.
2. Employment in the public sector is expected to amplify the risk of reduced work capacity resulting from biomechanical and psychosocial job exposures.
3. Working for a large employer is expected to mitigate the risk of reduced work capacity resulting from biomechanical job exposures but not that of psychosocial job exposures.
4. Workplace policies and practices to retain workers with reduced capacity are expected to mitigate the risk of reduced capacity resulting from biomechanical and psychosocial job exposures.

## **Methods**

### Data

This study uses register data obtained from a singular cohort of the Norwegian population, spanning the years 2005 to 2019, retrieved from by Statistics Norway and the Norwegian Welfare Administration (NAV). The registers encompass information on demographics, education, labor market participation, and welfare benefits. All registers are connected by anonymized unique individual identifiers, which include a linked employer-employee (LEE) identifier. Moreover, this article uses two recently developed JEMs: one for biomechanical job exposures and the other on psychosocial job exposures (Hermansen and Dahl 2022; Le et al. 2023). These matrices were derived from five iterations of the Norwegian living condition surveys (Statistics Norway 2024).

### Sample

The study sample was identified based on three eligibility criteria. First, individuals needed to have an active labor market record in the baseline year, 2010. Second, the sample was restricted to individuals aged 40 at baseline. This age group was selected because they have participated in the workforce for a considerable period following completion of their education, and have therefore likely accumulated job exposure over their labor market participation (likely 10–20 years). Third, individuals with a prior history of reduced work capacity in the five years leading up to baseline (2005 to 2009) were excluded. Individuals with a history of reduced work capacity were identified through two welfare benefits: work assessment allowance (WAA) and disability pensions (DP). WAA is a benefit for individuals with temporary reduced work capacity. To be eligible, recipients must have a reduced work capacity of at least 50%, or 30% for work related illness or injury (NAV 2024d). Additionally, work capacity must be improvable, either through (vocational) rehabilitation or through counseling through NAV (NAV 2024d). DP is a benefit for individuals with permanently reduced work capacity. To be eligible, work capacity must be reduced by at least 50%, or 30% for work related illness or injury (NAV 2024a). Furthermore, the recipient must have undergone treatment or counseling to improve work capacity, without result. Individuals can receive WAA and DP directly, or after exhausting the 52 weeks of paid sick leave if they are still unable to return to work. The net compensation decreases from 100% during sick leave to 66% under WAA or DP. Sick leave therefore reflects short-term health fluctuations, whereas WAA and DP reflect more sustained reductions in work capacity. The exclusion criterion was implemented to ensure that the sample comprised individuals with sufficient health to sustain employment over time. This can minimize the influence of preexisting health conditions that could lead to DP or WAA, allowing us to better capture the impact of job exposures on risk of reduced work capacity. Nevertheless, the inclusion of healthy individuals at the age of 40 may introduce healthy worker bias (Chowdhury et al. 2017; McMichael 1976), as the selected individuals are likely to be healthier than the broader population. The selection process resulted in a sample consisting of 37,439 observations.

### Variable operationalizations

#### Dependent variable

The outcome variable was defined as workers experiencing reduced work capacity during the follow up period (2010–2019). Reduced work capacity was defined as the receipt of either WAA or DP (see eligibility criteria in Sect. 2.2), and a dichotomous outcome variable was constructed to indicate the recipiency. These two welfare benefits were combined due to the strong association between the recipiency of WAA and subsequent DP, as 79% of new recipients of DP had transitioned from WAA (NAV 2024c). Individuals may receive WAA for a duration of up to three years, which may result in a potential lag in job exposure related to DP.

#### Job exposure variables

Biomechanical and psychosocial job exposures were defined using two newly developed and validated gender-specific JEMs (Hermansen 2024; Hermansen and Dahl 2022; Le et al. 2023). Both JEMs are based on five waves of the national representative Norwegian survey on living conditions (Statistics Norway 2024). The JEMs comprise 268 unique occupational groups classified according to the STYRK-08 system, which is the Norwegian adaptation of the ISCO-08 occupation classification system. This ensures consistent job exposure evaluations across the biomechanical and psychosocial domains. The Biomechanical Job Exposure Matrix (MJEM) incorporates eight physical exposures: heavy lifting (over 20 kg), working with hands above shoulder height, heavy physical work, neck flexion, squatting/kneeling, forward bending, awkward lifting, and standing/walking (Hermansen and Dahl 2022). In constructing the MJEM, individual-level exposure items were dichotomized to identify exposed and non-exposed workers. The cut-off value for exposure on each item was performing the activity for at least one-quarter of the working day (≥ 25%), except for standing/walking, which was cut-off at  ≥ 50% (Hermansen and Dahl 2022). At the occupational level, each exposure variable was defined as the proportion of individuals within each occupational group identified as exposed. The biomechanical index was constructed as the mean of the eight biomechanical exposures at the occupational level, theoretically ranging from 0 to 1. While a value of one suggests exposure to all eight mechanical exposures, no occupational code reported exposure to all eight simultaneously (min = 0.01, max = 0.66, SD = 0.14). For a detailed description of the biomechanical index, please see Hermansen and Dahl (2022).

The Job Strain Index (JSI) (Le et al. 2023) is based on Karasek’s Demand-Control Model (Karasek 1979), which is used to evaluate psychosocial job strain by examining the interaction between job control and job demand. The JSI consists of two components, the first being psychological demands encompassing four items: quantitative demands, conflicting ways of doing things, insufficient resources and contradictory requests. The second component is decision-latitude, comprising six items: decide how to go about the work, decide pace of work, important decisions, use skills, develop skills, and monotonous work. At the individual level, the exposure items were split at the median to identify those exposed and non-exposed (Le et al. 2023). At the occupational level, each exposure variable was defined as the proportion of individuals within each occupational group identified as exposed. Job demands and decision-latitude indices were computed as the mean of their respective exposure variables. The JSI was then calculated as the mean of the job demand and decision latitude indices, such that higher values reflect higher job demands and lower job control. Similar to the MJEM, the JSI ranges theoretically from 0 to 1 (min = 0.16, max = 0.48, SD = 0.06). For a detailed description of the construction of the JSI, please see Le et al. (2023).

Job exposure was assessed by constructing quintiles based on the two JEMs (Hermansen and Dahl 2022; Le et al. 2023), whereby the first quintile denotes the 20% of the least exposed individuals, and the fifth quintile denotes the 20% of the most exposed individuals. Certain individuals were employed in jobs that did not correspond to the JEM categories. For these individuals, occupational codes were imputed from the preceding year, contingent upon the presence of job-related income and employment status of the individuals. This procedure was conducted five times. If an individual still did not have a corresponding JEM group associated with their employment, they were excluded from the sample.

#### Individual characteristics

We incorporated several covariates recognized as factors that could influence the risk of reduced work capacity. We included a binary variable to indicate whether the individual had higher education (0 = no university degree, 1 = university degree). To represent an individual’s health condition, we constructed a sickness history variable that quantifies the total months of sick leave for each person over the five years leading up to baseline. The registry data only accounts for sickness absences covered by the government, thereby excluding shorter absences (less than 14 days) typically managed by employers (NAV 2024b). Additionally, binary variables for sex (male = 0, female = 1), marital status (unmarried = 0, married = 1), children (no = 0, yes = 1), and to indicate whether the individual is a first-generation immigrant (no = 0, yes = 1) were included. All individual characteristics were recorded at baseline.

#### Employer characteristics

The LEE records were used to construct several aggregated employer characteristics, drawing inspiration from Ulstein (2023). Employer size was defined following the EU definition: small (up to 50 employees), medium (ranging from 50 to up 250 employees), and large (exceeding 250 employees) (European Commission 2003). Micro and small employers were combined to avoid excessively small categories.

A proxy for the implementation of workplace policies to retain workers with reduced capacity were included, following Ulstein (2023) and Van Ooijen et al. (2021). This proxy was defined as the average duration of employment, measured in months, with the same employer after the receipt of the initial WAA or DP. The workplace policy proxy serves as an indicator of the extent to which employers successfully retain employees with reduced work capacity within the workplace. The proxy was classified into quartiles representing intervals of up to 6 months, from 6–18 months, from 18–24 months, and more than 24 months. Employers with fewer than two employees were excluded to mitigate bias arising from individual employment impacting the results (Ulstein 2023).

Four variables on the composition of the workplace were constructed. Age composition was defined as the proportion of employees aged 45 years or older at each employer. Wage composition was defined as the average wages of employees at each employer. Educational composition was defined as the proportion of employees with higher education within each employer. Additionally, a gender composition variable was created, indicating the fraction of female employees. Each of the four composition variables was categorized into quartiles, with the first quartile representing employers with the lowest values and the fourth quartile employers with the highest value.

Employer industry was based on the SN2007 system, which encompasses 21 categories. Industries of similar types were combined into seven categories to reduce complexity and mitigate the presence of excessively small categories. Finally, a sector variable was constructed to denote whether employees were employed in the public or private sector. All employer characteristics were recorded at baseline.

### Analytical strategy

To address our research questions, we employed a multi-step analytical strategy. First, we generated descriptive statistics to illustrate the distribution of individual and employer characteristics, as well as the proportion of individuals who experienced reduced work capacity during follow-up. Additionally, we summarized the proportion of individuals in each biomechanical and psychosocial exposure quintile across categories of the employer-level variables. Next, we estimated four Cox proportional hazard regression models (Blossfeld et al. 2019; Cleves et al. 2016). A key advantage of the Cox model is its semi-parametric nature, which does not require a predefined baseline hazard and makes fewer assumptions about the distribution of survival times. However, the model relies on the proportional hazard assumption, which must be validated.

To screen covariates for inclusion, we conducted univariate analyses. For continuous variables, we applied univariate Cox regression, while for categorical variables, we used the log-rank test and visual inspection of log cumulative hazard plots. Variables with a p-value below 0.25 were retained for multivariate modeling. The proportional hazard assumption was assessed both at the variable level and for the full model using log cumulative hazard plots, confirming that all covariates met the proportional hazard assumption and were suitable for inclusion in analysis.

Finally, four Cox proportional hazard models were estimated to assess the impact of biomechanical and psychosocial job exposures, and employer characteristics on the risk of reduced work capacity. The first model included all independent variables without interaction terms. The subsequent three models incorporated interaction terms between psychosocial and biomechanical exposures and each of the three employer characteristics: employer size, workplace policy proxy, and sector. All models were adjusted for both individual and employer-level covariates.

## Results

### Descriptive statistics

The descriptive statistics are presented in Table 1. The sample comprised 37,439 individuals, of whom 5,451 experienced reduced work capacity during follow-up. A majority of the individuals had lower education, were married, had children, and were not first-generation immigrants. Females, individuals with lower educational attainment, and those who were unmarried were more likely to experience reduced work capacity. Those who experienced reduced work capacity during follow-up had more sickness absences during the 5 years leading up to baseline (mean = 15.79, SD = 14.75), compared with the full sample (mean = 7.62. SD = 9.92). For higher levels of psychosocial and biomechanical exposures, a greater proportion of employees experienced reduced work capacity. Employers with lower mean wages, a lower proportion of highly educated employees, a higher proportion of women, and smaller size had a higher proportion of employees experiencing reduced work capacity.

**Table 1:** Descriptive statistics

Employer characteristics	% N	% Event	Individual characteristics	% N	% Event
*Sector*			*Sex*		
Public	60.38	14.29	Male	49.37	10.12
Private	39.62	14.97	Female	50.63	18.88
*Industry*					
Agriculture, forestry, and fishing	0.69	13.51	*Higher Education*		
Manufacturing, mining, energy supply	17.47	10.17	No	56.78	17.86
Construction	7.28	13.76	Yes	43.22	10.22
Transport, information, communication	14.51	15.74	*Marital status*		
Trade, finance, and real estate	10.99	13.92	Unmarried	46.98	16.59
Professional and public services	46.81	16.05	Married	53.02	12.76
Cultural, personal, and household	2.25	16.15	*Children*		
*Employer size*			No	13.68	14.20
Small	46.82	17.77	Yes	86.32	14.62
Medium	34.11	13.08	*First generation immigrant*		
Large	19.07	9.33	No	86.80	14.33
*Gender composition*			Yes	13.20	16.06
First	17.50	13.11	*Quintiles of psychosocial exposure*		
Second	32.74	10.99	First	11.94	11.70
Third	31.78	15.07	Second	24.92	13.52
Highest	17.99	21.56	Third	14.95	12.18
*Age composition*			Fourth	22.55	17.39
First	14.76	18.79	Highest	26.65	15.65
Second	34.92	14.53			
Third	40.36	13.14	*Quintiles of biomechanical exposure*		
Highest	9.96	14.26	First	19.26	8.90
*Wage composition*			Second	16.44	9.99
First	4.96	30.28	Third	16.94	13.74
Second	20.21	21.83	Fourth	24.71	20.11
Third	26.54	16.64	Highest	22.64	17.24
Highest	48.29	8.76			
*Educational composition*			Sickness history (mean)	7.62	15.79
First	5.12	21.80			
Second	31.44	15.63			
Third	26.90	16.50			
Highest	36.54	11.20			
*Workplace policy proxy*					
First	9.51	13.99			
Second	16.13	14.76			
Third	50.87	14.26			
Highest	23.39	15.29			
Total, N	37,439	5451		37,439	5451

Table 2 presents the distribution of observations across quintiles of biomechanical and psychosocial job exposures by employer size, workplace policy proxy, and sector. The quintiles represent increasing levels of exposure, with the highest quintile indicating the most exposed group. The highest level of biomechanical exposure was somewhat more common among employees in medium-sized firms (26.1%) than among those in small (20.8%) or large firms (21.0%), and among employees in the private sector (23.5%) compared with the public sector (21.4%). Employees in the highest quartile of the workplace policy proxy had a lower proportion in the highest biomechanical exposure quintile (17.1%) compared with the other quartiles (22.1%–25.1%). The highest level of psychosocial exposure was more prevalent among public-sector employees (47.8%) than among private-sector employees (12.8%). It was also slightly more common among employees in medium-sized firms (32.2%) than in small (21.9%) or large firms (28.3%), and among those in the third quartile of the workplace policy proxy (31.6%) compared with the other quartiles (16.2–24.4%).

**Table 2:** Distribution of employees across quintiles of biomechanical and psychosocial exposures by employer characteristics

Employer characteristics	Quintiles of biomechanical exposure, %					
	First	Second	Third	Fourth	Highest	Total N
Employer Size						
Small	14.5	14.5	19.4	30.8	20.8	17,529
Medium	21.2	16.1	17.2	19.4	26.1	12,772
Large	27.5	22.0	10.3	19.3	21.0	7138
Workplace policy proxy						
First	19.9	16.8	14.8	26.4	22.1	3560
Second	18.0	16.4	15.2	25.3	25.1	6038
Third	18.3	16.3	16.6	25.2	24.5	19,047
Highest	21.8	16.7	19.8	24.6	17.1	8794
Sector						
Public	15.9	13.1	21.8	27.8	21.4	22,607
Private	21.5	18.7	13.8	22.7	23.5	14,832

Employer characteristics	Quintiles of psychosocial exposure, %					
	First	Second	Third	Fourth	Highest	Total N
Employer Size						
Small	14.1	29.1	13.3	21.6	21.9	17,529
Medium	9.2	20.0	15.2	23.3	32.2	12,772
Large	6.3	23.4	18.5	23.4	28.3	7138
Workplace policy proxy						
First	15.5	27.8	17.6	22.8	16.2	3560
Second	12.7	23.4	17.1	26.2	20.5	6038
Third	9.0	22.3	14.3	22.8	31.6	19,047
Highest	12.1	30.4	13.9	19.3	24.4	8794
Sector						
Public	3.2	21.3	8.0	19.7	47.8	22,607
Private	16.0	27.3	19.5	24.4	12.8	14,832

### Results from analyses

In the first Cox proportional hazard model, estimated without interaction terms, psychosocial job exposure significantly increased the risk of reduced work capacity among employees in the third and highest quintiles compared with the least exposed group (please see supplementary material Table S1 for details). The HRs were 1.13 for both levels of psychosocial job exposure. Furthermore, the three highest levels of biomechanical job exposure significantly increased the risk of reduced work capacity in comparison to the least exposed group. The HRs ranged from 1.20 to 1.38 for the biomechanical job exposures (please see supplementary material Table S1 for details).

Table 3 presents the results from the Cox proportional hazard regression model, which includes interaction terms between employer size and job exposures. For main effects, the two highest quintiles of biomechanical exposure demonstrated significant associations with reduced work capacity, both with HRs of 1.22. Regarding employer characteristics, working for medium- and large-sized employers significantly decreased the risk of reduced work capacity, both with HRs of 0.52. For interaction terms, working for medium-sized employers exhibited significant interactions with the top two quintiles of biomechanical job exposure and the highest quintile of psychosocial job exposure, thereby increasing the risk of reduced work capacity. The HRs were 1.45 and 1.40 for the biomechanical exposure, respectively, and 1.41 for the psychosocial exposure. Furthermore, among employees working for large employers, significant interactions were observed between the third and highest quintiles of psychosocial job exposure, increasing the risk of reduced work capacity. The HRs were 1.63 and 1.86, respectively. For a calculation of total effects of the significant interactions, please see Table S2 in the supplementary material.

**Table 3:** Proportional Cox regression model with interaction with employer size and job exposures

Variable	HR	CI
*Quintiles of psychosocial exposure (reference first)*		
Second	0.96	0.84–1.11
Third	1.05	0.91–1.22
Fourth	1.04	0.90–1.20
Highest	0.96	0.83–1.11
*Quintiles of biomechanical exposure (reference first)*		
Second	0.99	0.85–1.16
Third	1.10	0.95–1.27
Fourth	1.22**	1.06–1.40
Highest	1.22**	1.06–1.42
*Sector (reference private)*		
Public	0.95	0.88–1.03
*Employer size (reference small)*		
Medium	0.52***	0.39–0.69
Large	0.52**	0.33–0.81
*Workplace policy proxy (reference first)*		
Second	1.12*	1.00-1.25
Third	1.14**	1.03–1.26
Highest	1.16**	1.04–1.29
Employer size * Psychosocial job exposure		
Medium– second	1.27	0.96–1.68
Medium– third	1.19	0.89–1.59
Medium * fourth	1.27	0.97–1.66
Medium * highest	1.41**	1.09–1.83
Large * second	1.31	0.83–2.06
Large * third	1.63*	1.04–2.57
Large * fourth	1.38	0.89–2.13
Large * highest	1.86**	1.21–2.86
Employer size * Biomechanical job exposure		
Medium * second	1.30*	1.00-1.68
Medium * third	1.25	0.98–1.60
Medium* fourth	1.45***	1.16–1.80
Medium * highest	1.40**	1.12–1.75
Large * second	0.88	0.65–1.19
Large * third	1.27	0.93–1.75
Large * fourth	1.00	0.75.1.32
Large * highest	1.02	0.78–1.33

HR = Hazard ratios, CI = 95% Confidence intervals, *p = .05, **p = .01, ***p = .001. Estimates are adjusted for individual and employer characteristics. *N* = 37 439

Table 4 presents the results of the Cox proportional hazard regression model that incorporates interaction terms between the workplace policy proxy and job exposures. For main effects, the second and highest quintiles of psychosocial job exposure and the fourth level of biomechanical job exposure yielded significant impacts on the risk of reduced work capacity, with HRs of 0.68, 0.68, and 1.51, respectively. Working for an employer with workplace policy proxy in the third quartile significantly decreased the risk of reduced work capacity compared to those in first quartile, with an HR of 0.68. Most interaction terms between the workplace policy proxy and quintiles of psychosocial job exposure significantly increased the risk of reduced work capacity, except for interactions involving employees in the second and highest quartile of workplace policy proxy and the third and fourth quintiles of psychosocial job exposure. The HRs ranged from 1.50 to 1.90. No statistically significant interactions were found between the workplace policy proxy and quintiles of biomechanical job exposure. For a calculation of the total effects of these significant interactions, please see Table S2 in the supplementary material.

**Table 4:** Proportional Cox regression model with interaction with workplace policy proxy and job exposures

Variable	HR	CI
*Quintiles of psychosocial exposure (reference first)*		
Second	0.68*	0.50–0.93
Third	0.82	0.59–1.14
Fourth	0.87	0.64–1.18
Highest	0.68*	0.49–0.95
*Quintiles of biomechanical exposure (reference first)*		
Second	1.02	0.70–1.48
Third	1.24	0.88–1.77
Fourth	1.51**	1.11–2.05
Highest	1.18	0.85–1.63
*Sector (reference private)*		
Public	0.95	0.88–1.03
*Employer size (reference small)*		
Medium	0.87***	0.81–0.93
Large	0.81***	0.73–0.90
*Workplace policy proxy (reference first)*		
Second	0.94	0.62–1.44
Third	0.68*	0.46–0.99
Highest	0.93	0.63–1.38
Workplace policy proxy * Psychosocial job exposure		
Second * second	1.53*	1.02–2.29
Second * third	1.37	0.90–2.09
Second * fourth	1.30	0.89–1.92
Second * highest	1.72*	1.13–2.61
Third * second	1.73**	1.21–2.46
Third * third	1.60*	1.10–2.33
Third * fourth	1.46*	1.03–2.06
Third * highest	1.90***	1.31–2.75
Highest * second	1.50*	1.04–2.17
Highest * third	1.29	0.87–1.92
Highest * fourth	1.15	0.80–1.65
Highest * highest	1.61*	1.09–2.37
Workplace policy proxy * Biomechanical job exposure		
Second * second	0.76	0.48–1.21
Second * third	0.80	0.52–1.24
Second * fourth	0.73	0.50–1.07
Second * highest	1.06	0.72–1.57
Third * second	1.16	0.77–1.75
Third * third	1.05	0.72–1.55
Third * fourth	1.01	0.72–1.41
Third * highest	1.29	0.91–1.83
Highest * second	1.06	0.69–1.63
Highest * third	0.92	0.62–1.38
Highest * fourth	0.86	0.60–1.22
Highest * highest	1.07	0.74–1.56

HR = Hazard ratios, CI = 95% Confidence intervals, *p = .05, **p = .01, ***p = .001. Estimates are adjusted for individual and employer characteristics. *N* = 37 439

Table 5 presents the results from the Cox proportional hazards regression model, which includes interaction terms between job exposures and sector of employment. The third, fourth, and highest quintiles of biomechanical job exposure were associated with an increased risk of reduced work capacity, relative to the least exposed. The HRs ranged between 1.29 and 1.47. The highest quintile of psychosocial job exposure was associated with increased risk of reduced work capacity, with an HR of 1.23. There were no significant differences in the risk of reduced work capacity between employees in the public and private sectors. Furthermore, none of the interaction terms yielded significant results.

**Table 5:** Proportional Cox regression model with interaction with sector and job exposures

Variable	HR	CI
*Quintiles of psychosocial exposure (reference first)*		
Second	0.99	0.87–1.12
Third	1.11	0.98–1.27
Fourth	1.13	0.99–1.28
Highest	1.23**	1.06–1.41
*Quintiles of biomechanical exposure (reference first)*		
Second	1.06	0.92–1.22
Third	1.29***	1.12–1.49
Fourth	1.39***	1.22–1.59
Highest	1.47***	1.28–1.69
*Sector (reference private)*		
Public	0.95	0.68–1.33
*Employer size (reference small)*		
Medium	0.88***	0.82–0.94
Large	0.81***	0.73–0.89
*Workplace policy proxy (reference first)*		
Second	1.11	1.00-1.24
Third	1.15**	1.04–1.27
Highest	1.16**	1.05–1.29
Sector * Psychosocial job exposure		
Public * second	1.17	0.83–1.63
Public * third	1.18	0.83–1.70
Public * fourth	1.01	0.73–1.41
Public * highest	0.94	0.68–1.31
Sector * Biomechanical job exposure		
Public * second	1.06	0.84–1.34
Public * third	0.86	0.69–1.08
Public * fourth	0.96	0.79–1.17
Public * highest	0.88	0.72–1.09

HR = Hazard ratios, CI = 95% Confidence intervals, *p = .05, **p = .01, ***p = .001. Estimates are adjusted for individual and employer characteristics. *N* = 37 439

## Discussion

This study examined the impact of biomechanical and psychosocial job exposures on risk of reduced work capacity in a cohort of Norwegian workers, focusing on whether the impact varied by employer sector, organizational size, and workplace policies. In line with previous research, both biomechanical and psychosocial job exposures were associated with increased risk of reduced work capacity (d’Errico et al. 2021; Ervasti et al. 2019; Niedhammer et al. 2021). These findings suggest that moderate to high levels of job-related exposure can contribute to reduced work capacity and, in some cases, temporary or permanent labor market exit. These findings, together with previous research, highlight the importance of workplace interventions that can reduce the impact of such exposures on individual work capacity to sustain and improve worker health, ultimately contributing to prolonging working life trajectories.

There was some variation in the impact of biomechanical and psychosocial job exposures according to employer characteristics. In line with previous research, working for a medium-sized or large employer while experiencing higher levels of psychosocial job exposure (the highest quintile, and third and highest quintile, respectively) significantly increased the risk of reduced work capacity (Knani et al. 2021; Lai et al. 2015; Sørensen et al. 2007). Medium and large employers may encounter greater difficulties in fostering a positive psychosocial work environment due to size—for example, high quantitative demands and lower job control—resulting from standardized processes and bureaucratic structures (Karasek 1979; Storey et al. 2010). This can lead to an amplification of psychosocial job exposures for those working for medium-sized and large employers. Contrary to our empirical expectations, working for a medium-sized employer while experiencing higher levels of biomechanical job exposures (second, fourth and highest quintiles), significantly increased the risk of reduced work capacity (Knani et al. 2021; Sørensen et al. 2023). Medium-sized employers may face difficulties in maintaining a healthy work environment due to their position between the informal structures typical of small employers and the formalized systems of large organizations. This could lead to the amplification of biomechanical exposures. The empirical expectations are therefore partially met for employer size.

Contrary to our expectations, we found that those who worked for employers with more extensive workplace policies aimed at retaining workers with reduced capacity (second through fourth quartiles), and who were simultaneously exposed to higher levels of psychosocial job exposure (ranging from the second to the highest quintile, depending on the policy proxy), faced a significantly higher risk of reduced work capacity. This is inconsistent with previous research, which identified workplace policies as protective factors for continued employment (Hulsegge et al. 2022; Kersten et al. 2023; Ulstein 2023; Van Ooijen et al. 2021). However, previous studies estimated the impact of workplace policies on continued or sustained employment for workers already experiencing reduced capacity, rather than on their preventive impact among healthy workers. The associations identified in the current study suggest that organizational policies designed to support workers with reduced capacity may have limited effectiveness in reducing the risk posed by psychosocial job exposure among healthy employees. At the same time, such policies may still play a valuable role in supporting individuals who already experience reduced work capacity. To effectively reduce the impact of psychosocial job strain on the risk of reduced work capacity, it may be necessary to implement distinct workplace policies that improve psychosocial working conditions, with tailored approaches targeting both healthy workers and those with reduced capacity. Moreover, in contrast to our expectations, the main effect of psychosocial job exposure (second and highest quintile) was associated with a significantly lower risk of reduced work capacity. This pattern may reflect healthy worker bias (Chowdhury et al. 2017; McMichael 1976). Occupations characterized by higher psychosocial demands—often high-pressure roles—are typically held by relatively healthy workers, as individuals with existing health problems are less likely to enter or remain in such positions. Consequently, psychosocial exposure may appear protective, although this is likely due to selection rather than a true protective impact.

No significant differences were found in the impact of psychosocial and biomechanical job exposures on the risk of reduced work capacity between private and public employers; in the impact of biomechanical exposure between large and small employers; or in the impact of biomechanical exposure in relation to implemented workplace policies. This is contrary to the empirical expectations, and indications from previous research for public sector employers (Albertsen et al. 2007; Hartikainen et al. 2022; Sørensen et al. 2023), and large employers (Fenn and Ashby 2004; Sørensen et al. 2007). The lack of variation suggests that, for certain employer characteristics, the negative impact of biomechanical and psychosocial exposures is relatively consistent—neither intensified nor mitigated. This consistency may present an advantage for intervention strategies designed to reduce the impact of biomechanical and psychosocial exposures, as it can support the implementation of more universal measures across different types of employers within occupations that share similar exposure profiles. With the right implementation and follow up, such interventions can reduce the negative impact of biomechanical and psychosocial exposures on risk of reduced work capacity, contributing to a sustainable and healthy workforce and preventing early labor market exits.

### Strengths and limitations

This study has several strengths. It used high-quality Norwegian registry data, allowing for complete national coverage and long-term follow-up of workers at age 40 while minimizing missing information and attrition typical in surveys. A ten-year follow-up enabled robust estimation of the risk of reduced work capacity, including lagged effects. To reduce confounding, individuals with a prior history of reduced work capacity were excluded. Biomechanical and psychosocial exposures were assessed using two validated JEMs, providing a consistent, standardized evaluation of job-related risks when linked to registry data (Hermansen and Dahl 2022; Le et al. 2023; Peters 2020).

Despite the exclusion of individuals with prior history of reduced work capacity, it is not possible to eliminate unobserved confounders, and individual health likely affected the incidence of reduced work capacity. Moreover, causal inferences regarding the impact of biomechanical and psychosocial job exposures on the risk of reduced work capacity cannot be drawn from this study. Prior exposures and pre-existing health conditions likely influenced which individuals remained in the workforce by age 40, potentially introducing selection and healthy worker biases into the estimates (Chowdhury et al. 2017; McMichael 1976). Exposure levels in this study were assessed only for the job held at age 40, which may underestimate individual’s true exposure. A single time point may not accurately capture an individual’s full work history or the exposure accumulated over time. By assuming static exposure from just one job, the approach overlooks variation and accumulation across different occupations. To improve accuracy, future research should incorporate cumulative exposure measures that account for an individual’s entire work trajectory. The use of exposure matrices may also introduce misclassification, as they assume uniform exposure levels for all individuals with the same occupational codes, ignoring personal and task variation within occupations (Peters 2020). A final limitation of using JEMs in the form of indexes is that information on specific exposures (e.g., heavy lifting or quantitative job demands) is lost. Using composite JEM measures means that the observed impact of biomechanical or psychosocial exposures cannot be attributed to underlying components, limiting the ability to identify which exposures are driving the associations. Such specificity is often crucial for designing and implementing targeted workplace interventions, as different exposures may require distinct preventive strategies. A way forward for future research could be to investigate whether the impact of specific biomechanical and psychosocial exposures vary with employer characteristics, to facilitate the design of targeted intervention strategies.

## Conclusion

This study contributes to evidence on the impact of biomechanical and psychosocial job exposures on risk of reduced work capacity. Using high-quality registry data and validated exposure matrices, we found that moderate to high levels of job-related exposures were associated with an increased risk of reduced work capacity. While employer characteristics such as size and organizational policies showed some variation in moderating these effects, the findings suggest that such contextual factors do not consistently mitigate or amplify the negative impact of job exposures on risk of reduced work capacity. Particularly, organizational policies intended to retain workers with reduced capacity may have limited preventive effects for healthy employees, though they may still play an important role in supporting those already experiencing reduced work capacity. These findings highlight the need for broader workplace interventions to reduce harmful exposures, particularly in high-risk occupations. Future research should prioritize the development of cumulative exposure measures and further explore how organizational practices can be designed to protect both vulnerable and healthy workers across different employment contexts.

## Supplementary Information

Below is the link to the electronic supplementary material.

Supplementary Material 1

## Data Availability

The data used in this study are confidential and cannot be shared publicly.
