# Supplementary material for: Job exposures, employer characteristics, and risk of reduced work capacity: a 10-year cohort study of Norwegian workers
Source: Int Arch Occup Environ Health. 2026 Jan 3;99(1):6. doi: 10.1007/s00420-025-02195-y (PMC12764626; doi:10.1007/s00420-025-02195-y)
Supplement: Supplementary file 1 — Supplementary Material 1 [file 420_2025_2195_MOESM1_ESM.docx]

Supplementary material

Table S1: Proportional Cox regression

| Employer characteristics | HR | CI | Individual characteristics | HR | CI |
| --- | --- | --- | --- | --- | --- |
| Sector (reference private) |  |  | Sex (reference male) |  |  |
| Public | 0.95 | 0.88-1.03 | Female | 1.64*** | 1.52-1.77 |
| Industry (reference: Agriculture, forestry, and fishing) |  |  | Higher Education (reference no) |  |  |
| Manufacturing, mining, energy supply | 1.10 | 0.78-1.54 | Yes | 0.67*** | 0.62-0.72 |
| Construction | 1.24 | 0.87-1.76 | Marital status (reference unmarried) |  |  |
| Transport, information, communication | 1.20 | 0.85-1.69 | Married | 0.79*** | 0.75-0.84 |
| Trade, finance, and real estate | 1.34 | 0.95-1.90 | Children (reference no) |  |  |
| Professional and public services | 1.28 | 0.91-1.81 | Yes | 0.94 | 0.86-1.02 |
| Cultural, personal, and household | 1.25 | 0.85-1.82 | First generation immigrant (reference no) |  |  |
| Employer size |  |  | Yes | 1.13** | 1.06-1.24 |
| Medium | 0.87*** | 0.81-0.93 |  |  |  |
| Large | 0.81*** | 0.74-0.90 | Psychosocial job exposure (reference first) |  |  |
| Gender composition (reference first) |  |  | Second | 1.03 | 0.92-1.16 |
| Second | 0.86** | 0.77-0.95 | Third | 1.13* | 1.01-1.28 |
| Third | 0.80*** | 0.70-0.91 | Forth | 1.11 | 0.99-1.25 |
| Highest | 0.78*** | 0.68-0.90 | Highest | 1.13* | 1.00-1.27 |
| Age composition (reference first) |  |  | Biomechanical job exposure (reference first) |  |  |
| Second | 0.93 | 0.86-1.01 | Second | 1.07 | 0.95-1.19 |
| Third | 0.90* | 0.83-0.99 | Third | 1.20*** | 1.08-1.34 |
| Highest | 0.92 | 0.82-1.03 | Forth | 1.38*** | 1.24-1.53 |
| Wage composition (reference first) |  |  | Highest | 1.38*** | 1.24-1.54 |
| Second | 0.71*** | 0.64-0.79 |  |  |  |
| Third | 0.62*** | 0.56-0.69 | Sickness history | 1.03*** | 1.03-1.03 |
| Highest | 0.42*** | 0.37-0.47 |  |  |  |
| Educational composition (reference first) |  |  |  |  |  |
| Second | 0.90 | 0.80-1.00 |  |  |  |
| Third | 0.78*** | 0.69-0.88 |  |  |  |
| Highest | 0.85* | 0.74-0.97 |  |  |  |
| Workplace policy proxy (reference first) |  |  |  |  |  |
| Second | 1.10 | 0.99-1.23 |  |  |  |
| Third | 1.14* | 1.03-1.25 |  |  |  |
| Highest | 1.15** | 1.04-1.28 |  |  |  |

HR=Hazard ratios, CI= 95% Confidence intervals, *p= .05, **p= .01, ***p= .001. Estimates are adjusted for individual and employer characteristics. N=37 439

Table S2. Calculation of total effects for significant interactions

| Variable combination | Main Effect 1 | Main Effect 2 | Interaction HR | Total HR |
| --- | --- | --- | --- | --- |
| Employer size = medium, job strain level = 5 | 0.52*** | 0.96 | 1.41 ** | 0.70 |
| Employer size = large, job strain level = 3 | 0.52*** | 1.05 | 1.63* | 0.89 |
| Employer size = large, job strain level = 5 | 0.52*** | 0.96 | 1.86** | 0.93 |
| Employer size = medium, biomechanical level = 2 | 0.52*** | 0.99 | 1.30* | 0.67 |
| Employer size = medium, biomechanical level = 4 | 0.52*** | 1.22** | 1.45 *** | 0.92 |
| Employer size = medium, biomechanical level = 5 | 0.52*** | 1.22** | 1.40* | 0.89 |
| Workplace policy proxy = 2, job strain level = 2 | 0.94 | 0.68* | 1.53 * | 0.98 |
| Workplace policy proxy = 2, job strain level = 5 | 0.94 | 0.68* | 1.72* | 1.10 |
| Workplace policy proxy = 3, job strain level = 2 | 0.68* | 0.68* | 1.73** | 0.80 |
| Workplace policy proxy = 3, job strain level = 3 | 0.68* | 0.82 | 1.60* | 0.89 |
| Workplace policy proxy = 3, job strain level = 4 | 0.68* | 0.87 | 1.46* | 0.86 |
| Workplace policy proxy = 3, job strain level = 5 | 0.68* | 0.68* | 1.90*** | 0.88 |
| Workplace policy proxy = 4, job strain level = 2 | 0.93 | 0.68* | 1.50* | 0.95 |
| Workplace policy proxy = 4, job strain level = 5 | 0.93 | 0.68* | 1.61* | 1.02 |

All coefficients in terms of HR=Hazard ratios. Total HR = main effect 1 x main effect 2 x interaction HR.
